# Multimodal imaging features of primary breast epithelial–myoepithelial carcinoma: a case report and literature review

**DOI:** 10.3389/fonc.2026.1845855

**Published:** 2026-07-01

**Authors:** Peilin Sha, Yunhao Luo, Xiangzhu Wang, Yanhong An, Jia Jia, Xue Shi, Xin Li, Mengke Xu, Huiyu Ge, Zexing Yu

**Affiliations:** 1 The Third Affiliated Hospital of Beijing University of Chinese Medicine, Beijing, China; 2 School of Medical and Life Sciences, Chengdu University of Traditional Chinese Medicine, Chengdu, China; 3 Dongzhimen Hospital, Beijing University of Chinese Medicine, Beijing, China; 4 Beijing Chaoyang Hospital, Capital Medical University, Beijing, China

**Keywords:** breast epithelial-myoepithelial carcinoma, case report, contrast-enhanced ultrasound, multimodal imaging, shear-wave elastography

## Abstract

Breast epithelial–myoepithelial carcinoma (EMC) is a rare biphasic malignant tumor composed of both epithelial and myoepithelial components, accounting for less than 0.5% of all breast tumors. Owing to its extremely low incidence, the imaging characteristics of EMC have not been fully described, particularly with regard to emerging ultrasound techniques, such as shear-wave elastography (SWE) and contrast-enhanced ultrasound (CEUS). Herein, we report the case of a 70-year-old woman with breast EMC. The multimodal imaging findings, including digital mammography, computed tomography (CT), magnetic resonance imaging (MRI), conventional ultrasound (grayscale and color Doppler), SWE, and CEUS, are systematically presented and analyzed in correlation with the pathological findings. This report aims to improve clinical understanding of the imaging features of EMC.

## 1 Introduction

Adenomyoepithelioma (AME) of the breast is a biphasic tumor composed of both glandular epithelial and myoepithelial cells of the mammary ducts and was first systematically described by Hamperl in 1970 (1). According to the fifth edition of the World Health Organization (WHO) Classification of Tumors of the Breast (2019), AME can be categorized as either benign or malignant. When malignant transformation involves both epithelial and myoepithelial components, the tumor is defined as epithelial–myoepithelial carcinoma (EMC) (2). EMC occurs predominantly in the salivary glands, particularly in the parotid gland (3), whereas primary EMC of the breast is extremely rare. As of 2022, approximately 150 cases have been reported in the English literature (4).

The clinical and imaging manifestations of breast EMC are non-specific and may overlap with those of invasive ductal carcinoma, benign AME, and metaplastic carcinoma. Previously reported imaging data on breast EMC have mostly been limited to single-case reports and have mainly focused on mammography, conventional ultrasonography, MRI, or metastatic evaluation. In recent years, shear-wave elastography (SWE) and contrast-enhanced ultrasound (CEUS) have been increasingly used in the evaluation of breast lesions. SWE can quantitatively assess lesion stiffness and stiffness heterogeneity, whereas CEUS can dynamically evaluate microvascular perfusion, enhancement patterns, perfusion defects, vascular morphology, and time–intensity curve parameters (5, 6). However, systematic descriptions of SWE and CEUS findings in this rare tumor remain extremely limited, and the potential value of SWE and CEUS in the preoperative assessment of breast EMC has not yet been established.

In this report, we describe a case of primary breast epithelial–myoepithelial carcinoma and present its multimodal imaging findings, including mammography, CT, MRI, conventional ultrasonography, SWE, and CEUS, in correlation with histopathological and immunohistochemical findings. Compared with previous reports, the main value of the present case lies in its relatively comprehensive multimodal imaging assessment, particularly the additional SWE and CEUS findings demonstrating stiffness heterogeneity and intratumoral perfusion heterogeneity. This case aims to add to the limited imaging literature on breast EMC and to illustrate the potential role of multimodal ultrasound in the preoperative evaluation of rare breast tumors with atypical or diagnostically challenging conventional imaging findings.

## 2 Case presentation

This study was approved by the Ethics Committee of Beijing Chaoyang Hospital, Capital Medical University (Approval No. 2026-Ke-346). A 70-year-old female patient presented to Beijing Chaoyang Hospital after a lump was detected in her right breast during a routine physical examination. A clinical examination revealed a palpable mass of approximately 1.0 cm in the 12 o’clock position of the right breast; the mass was firm, poorly mobile, with indistinct borders, and non-tender. No obvious mass was palpable in the left breast, and no enlarged lymph nodes were detected in the bilateral axillary or supraclavicular regions. Laboratory tests revealed no significant specific abnormalities. Only the complete blood count showed a mildly elevated white blood cell count (9.65 × 10^9^/L). Biochemical tests indicated elevated levels of lactate dehydrogenase (294 U/L) and α-hydroxybutyrate dehydrogenase (239 U/L), with mildly decreased total protein and albumin levels. Coagulation studies showed mildly elevated levels of fibrinogen degradation products and D-dimer. Among tumor markers, CYFRA21–1 was elevated to 8.00 ng/mL; no other markers showed significant abnormalities.

**Figure 1:**
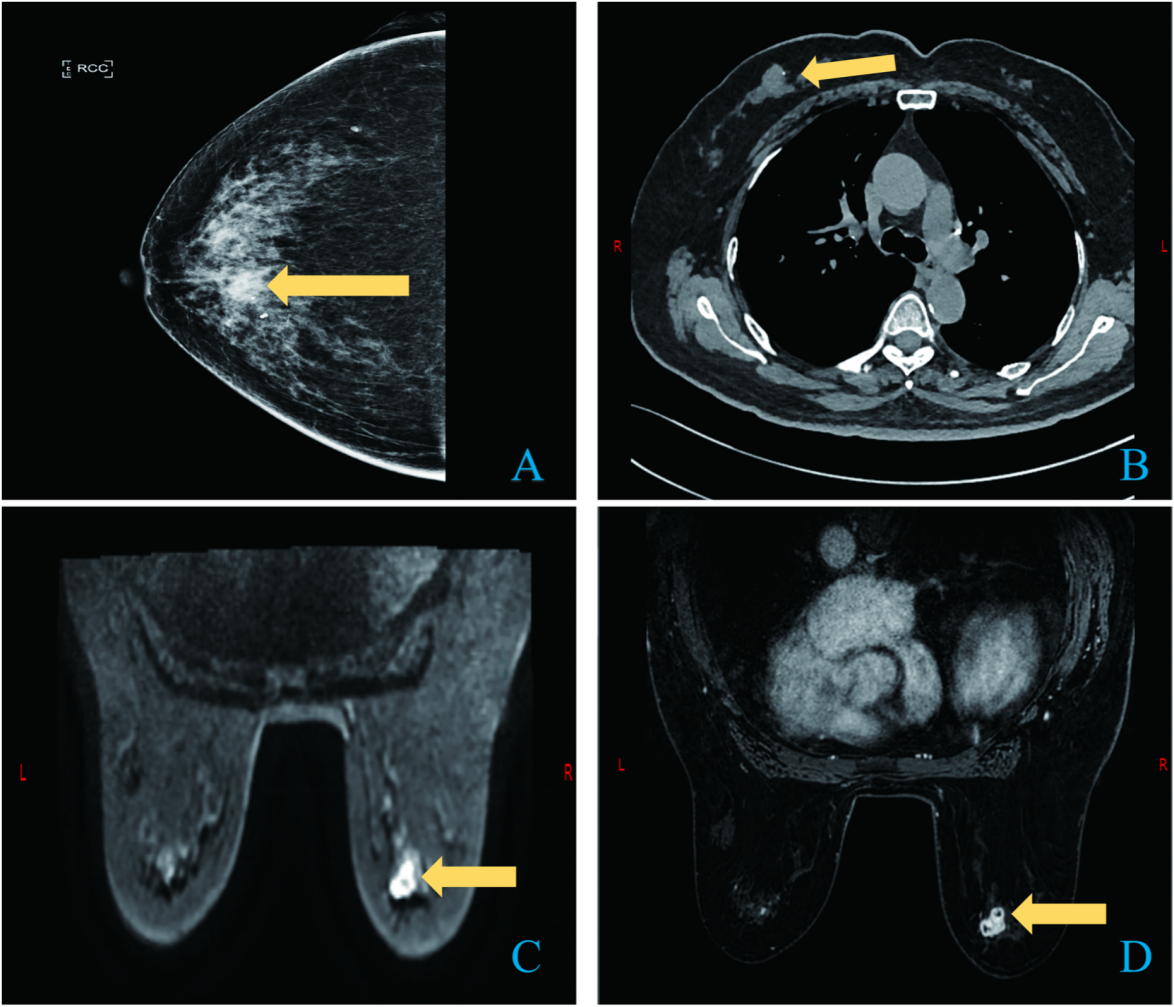
Multimodal imaging of the right breast lesion **(A–D)**. **(A)** Mammography shows a nodular opacity in the upper quadrant of the right breast. **(B)** Non-contrast chest CT shows a right breast nodule with peripheral calcifications. **(C)** Breast MRI DWI shows a hyperintense lesion in the upper inner quadrant of the right breast. **(D)** DCE-MRI shows marked enhancement with a washout-type time–signal intensity curve. Yellow arrows indicate the lesion.

The patient underwent multimodal imaging studies, including digital mammography, CT, MRI, conventional ultrasound, shear-wave elastography, and contrast-enhanced ultrasound. Digital mammography (**Figure 1A**) revealed a nodular opacity in the upper quadrant of the right breast, measuring approximately 1.0 cm × 1.4 cm, with an irregular shape and poorly defined margins; both breasts exhibited an irregular dense glandular pattern (ACR Type C). Non-contrast chest CT (**Figure 1B**) revealed a nearly circular nodular lesion in the right breast, measuring approximately 17 mm × 14 mm, with punctate calcifications at the margins; no definite metastatic lesions were observed in either lung. These findings suggested a space-occupying lesion in the right breast, but the presentation lacked specificity, and further ultrasound examination was recommended.

**Figure 2:**
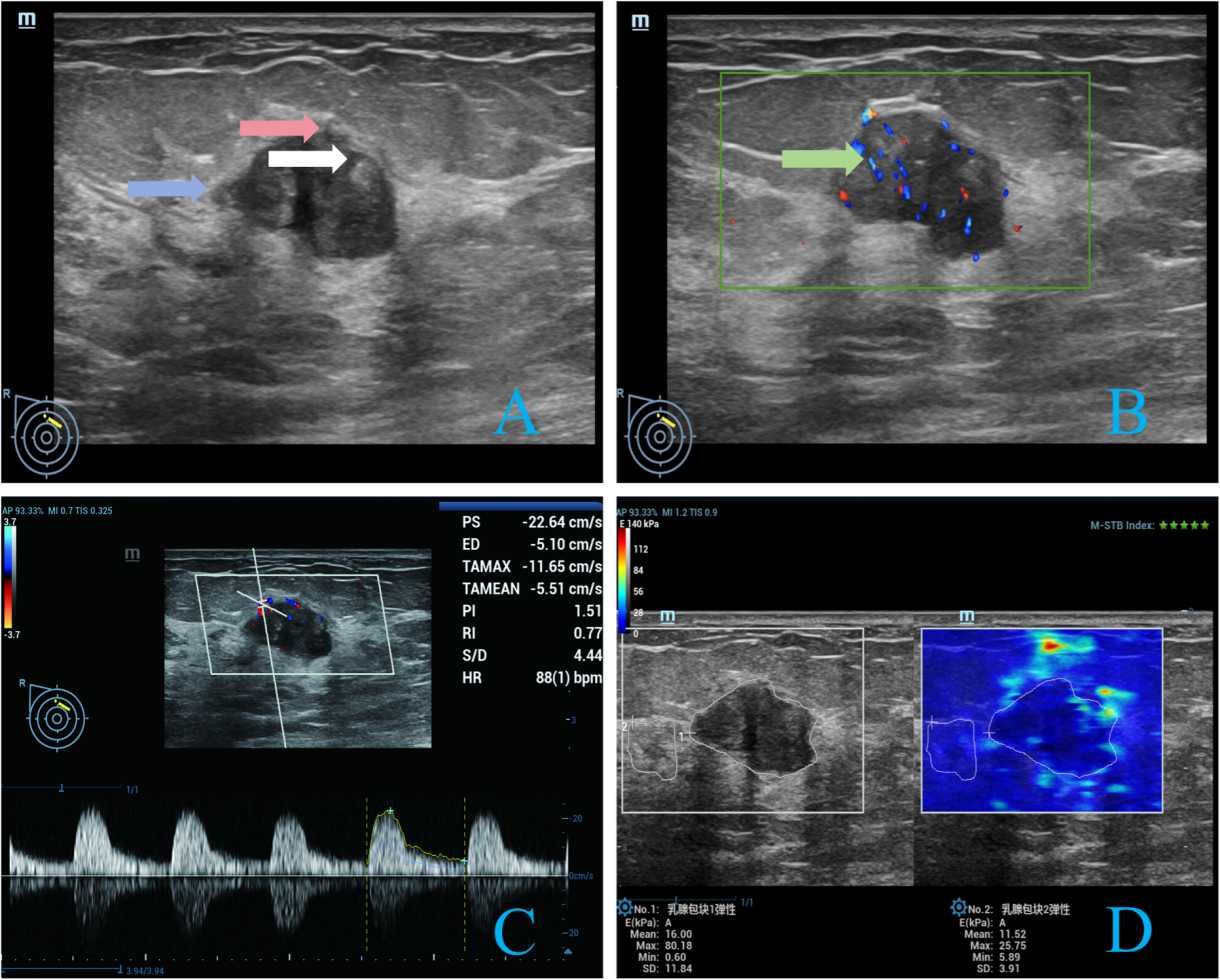
Multimodal ultrasound findings of the breast lesion **(A–D)**. **(A)** Grayscale ultrasound shows an irregular hypoechoic lesion with indistinct margins, focal microlobulations, spiculated margins, and peripheral punctate echogenic foci. **(B)** Color Doppler imaging shows feeding vessels entering the lesion and internal vascular signals. **(C)** Pulsed-wave Doppler demonstrates an arterial waveform within the lesion. **(D)** SWE demonstrates heterogeneous stiffness distribution within the lesion, with increased stiffness compared with the adjacent normal glandular tissue. Blue arrows indicate microlobulations, pink arrows indicate spiculated margins, white arrows indicate punctate echogenic foci, and green arrows indicate vascular signals.

Conventional ultrasound, shear-wave elastography (SWE), and contrast-enhanced ultrasound (CEUS) were performed by a chief physician with more than 20 years of experience in breast ultrasound, breast elastography, and contrast-enhanced ultrasound. All ultrasound examinations were performed using a Mindray Resona R9S ultrasound system equipped with an L15-3WU linear-array transducer. SWE was performed in shear-wave elastography mode, with elasticity parameters displayed in kPa and an elasticity scale of 0–140 kPa. The main SWE settings included an imaging depth of 3.5 cm, gain of 49, frame rate of 6 frames/s, dynamic range of 140, and iClear+ level 2. During image acquisition, the transducer was gently placed on the skin surface to avoid excessive compression. The ROI was placed within the solid component of the lesion, and adjacent normal glandular tissue was measured as a reference. Obvious cystic change, necrosis, calcification, and artifacts were avoided as much as possible. Measurements were obtained after the elastography image had stabilized. Conventional ultrasound (**Figure 2**) revealed a hypoechoic lesion at the 12 o’clock position of the right breast, measuring approximately 1.5 cm × 1.2 cm, with indistinct borders and an irregular shape. Some edges showed fine lobulations and spicules, while others exhibited a small, irregularly thickened hyperechoic halo. The internal echogenicity was heterogeneous, with a few irregular hypoechoic to anechoic areas; multiple punctate hyperechoic foci were visible at the margins, and mild posterior acoustic enhancement was observed. Color Doppler imaging showed large feeding vessels penetrating the margin of the lesion; no circumferential flow signals were observed. Abundant blood flow signals were visible within the lesion, with an arterial-like flow spectrum detectable. The peak flow velocity was 22.64 cm/s, the end-diastolic velocity was 5.10 cm/s, and the resistance index was 0.77. The lesion had a mean elastic modulus of 16.00 kPa, a maximum elastic modulus of 80.18 kPa, and a minimum elastic modulus of 0.60 kPa. The adjacent normal glandular tissue had a mean elastic modulus of 11.52 kPa, a maximum elastic modulus of 25.75 kPa, and a minimum elastic modulus of 5.89 kPa. These findings indicated that the lesion was stiffer than the adjacent normal glandular tissue and showed greater internal stiffness heterogeneity.

**Figure 3:**
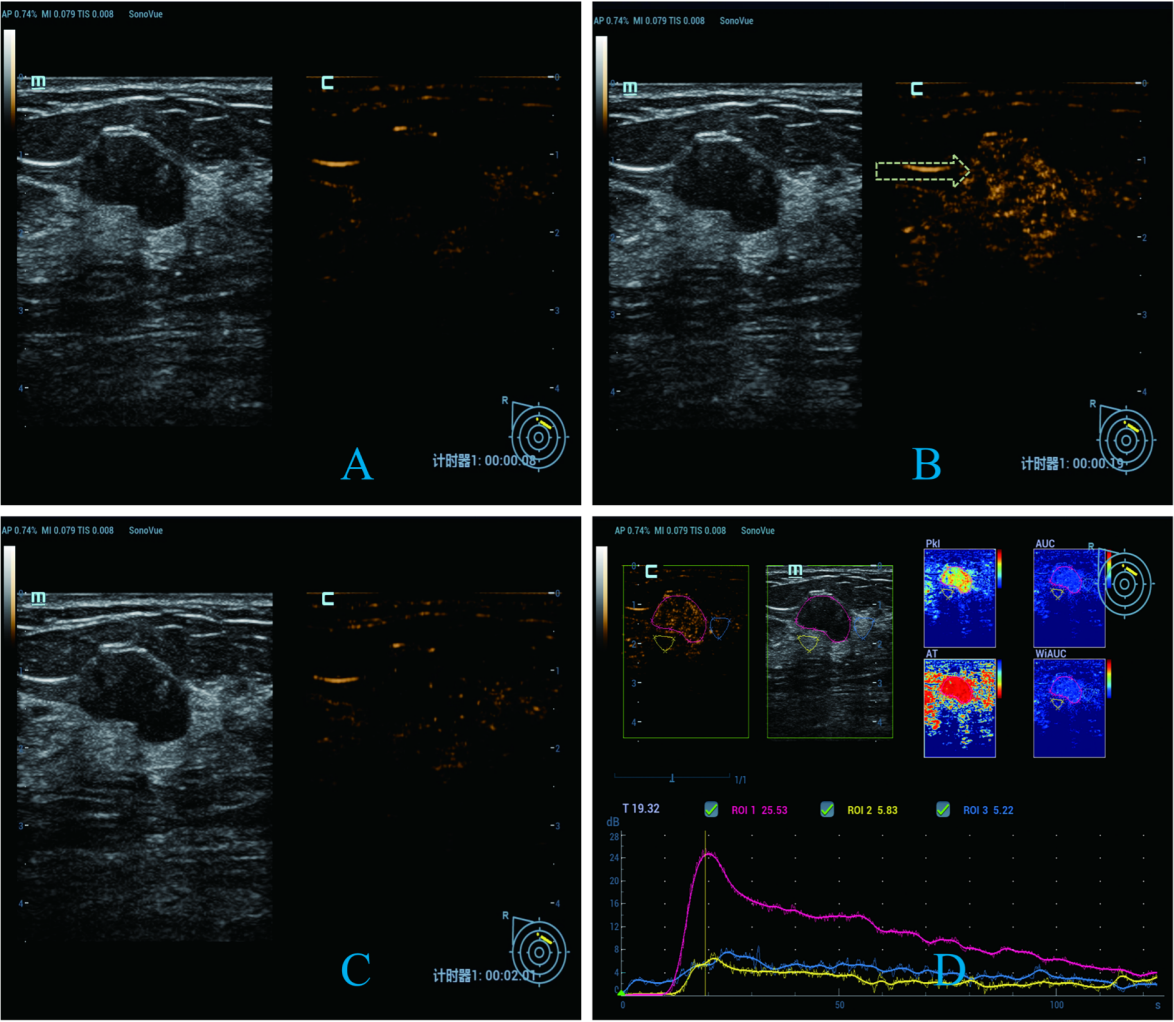
Contrast-enhanced ultrasound (CEUS) images and time–intensity curve (TIC) analysis **(A–D)**. **(A)** Early wash-in phase. **(B)** Peak enhancement phase, showing heterogeneous enhancement with linear hyperenhancing structures and hypo- to non-enhancing areas. **(C)** Late phase, showing contrast washout. **(D)** Time–intensity curves of the lesion and adjacent glandular tissues. Green dashed arrows indicate linear hyperenhancing areas within the lesion.

CEUS was performed using a contrast-specific imaging mode with a low mechanical index of 0.079. The main CEUS settings included an imaging depth of 4.5 cm, contrast gain of 38, frame rate of 15 frames/s, contrast dynamic range of 110, tissue image gain of 60, tissue image dynamic range of 115, and iClear+ level 2. After a bolus injection of 4.8 mL SonoVue through the left antecubital vein, dynamic contrast-enhanced images were continuously stored for 180 s. The imaging parameters remained unchanged during acquisition. For TIC analysis, the ROI was placed within the enhancing solid component of the lesion, while large vessels, calcifications, acoustic shadowing, non-enhancing areas, and obvious artifacts were avoided. As shown in the patient’s contrast-enhanced ultrasound (**Figure 3**), the lesion began to enhance at 8 seconds, with near-simultaneous enhancement of the margins and interior; peak enhancement was reached at 19 seconds, with an overall peak intensity of 25.53 dB, and no significant expansion of the enhancement area was observed. Internal enhancement of the lesion was markedly heterogeneous, with multiple linear areas of high enhancement. These linear areas of high enhancement exhibited a slightly map-like distribution, while the interior showed low to no enhancement; the contrast agent demonstrated a rapid uptake and washout pattern overall. Based on the combined findings of conventional ultrasound, SWE, and CEUS, the lesion was classified as BI-RADS 4C, and a needle biopsy was recommended.

The results of the ultrasound-guided fine-needle aspiration biopsy suggested invasive breast cancer. Following the biopsy result suggestive of malignancy, breast MRI was performed for further staging evaluation. Breast MRI (**Figures 1C, D**) revealed a lobulated mass in the upper inner quadrant of the right breast, approximately 1.8 cm in length, with high signal intensity on both T2-weighted and diffusion-weighted images. Dynamic contrast-enhanced imaging showed marked enhancement of the lesion, with a washout pattern on the time–signal intensity curve. No clearly enlarged lymph nodes were observed in either axilla. The mass in the right upper inner quadrant was classified as BI-RADS Category 6.

**Figure 4:**
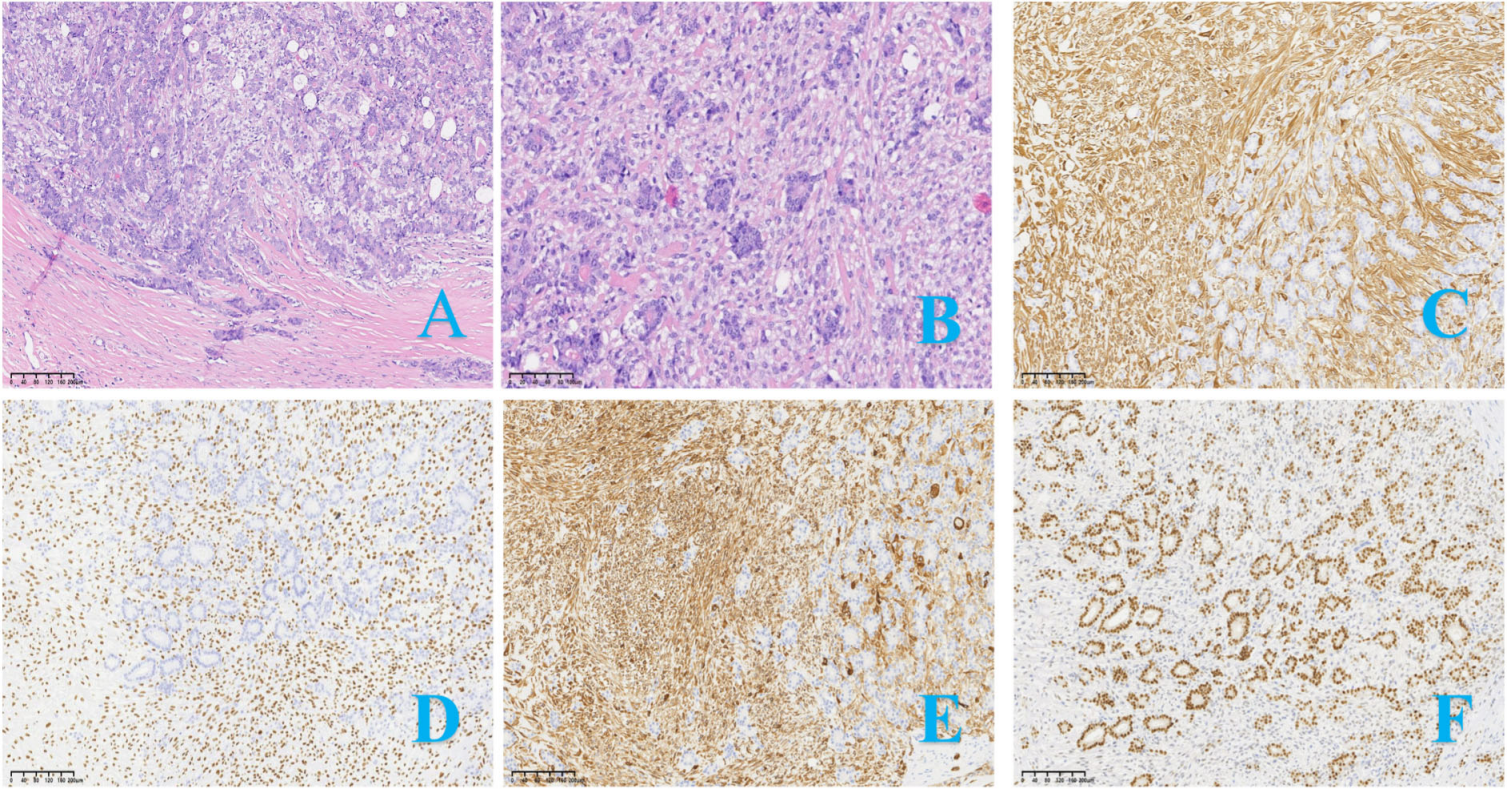
Histopathological and immunohistochemical findings of the lesion **(A–F)**. **(A, B)** Hematoxylin and eosin staining shows a cellular tumor with cytologic atypia, scattered mitotic figures, and infiltrative growth at the periphery. **(C)** CK5/6 positivity. **(D)** P63 positivity in the myoepithelial component. **(E)** CK14 positivity. **(F)** GATA3 positivity.

The final pathological examination of the postoperative surgical specimen revealed an adenomyoepithelial tumor of the right breast with marked cytologic atypia, mitotic activity of approximately 3 mitoses per 10 high-power fields in the most active areas, and infiltrative growth at the periphery (**Figures 4A, B**). These findings were consistent with atypical adenomyoepithelioma with malignant transformation, namely epithelial–myoepithelial carcinoma. The surgical margins were negative. The surrounding breast tissue showed usual ductal epithelial hyperplasia. No metastatic carcinoma was identified in the right sentinel lymph nodes (0/4). Immunohistochemistry showed positivity for CK5/6, CK14, P63, EGFR, and GATA3, and partial weak positivity for P53; representative positive staining for CK5/6, P63, CK14, and GATA3 is shown in **Figures 4C–F**. ER showed moderate positivity in approximately 40% of tumor cells, PR was negative, HER2 was scored as 2+, AR showed weak positivity in approximately 25% of tumor cells, and the Ki-67 proliferation index was approximately 10% in the hypercellular areas.

At the 6-month postoperative follow-up, the patient was in good general condition. Follow-up breast ultrasound showed postoperative changes in the right breast, with no clinical or ultrasonographic evidence suggestive of local recurrence or axillary lymph node metastasis.

## 3 Discussion

Breast epithelial–myoepithelial carcinoma is a rare biphasic malignant tumor that predominantly occurs in women over 60 years of age. Clinically, it often presents as a long-standing breast mass that undergoes rapid enlargement within a short period, typically as a painless, firm, solitary nodule with a tendency for infiltrative growth. A small proportion of cases may develop regional lymph node or distant metastasis (7, 8). Local recurrence may also occur, and complete surgical excision with negative margins remains the main treatment strategy (9). The present patient was a 70-year-old woman, consistent with the typical age distribution of EMC. Therefore, newly detected breast nodules in middle-aged and elderly women should raise suspicion for malignancy. In this case, the right breast nodule was incidentally detected during routine physical examination. On specialist examination, the nodule was firm and non-tender, but the clinical findings were not specific. The significance of this case lies in its relatively comprehensive presentation of the multimodal imaging features of breast EMC, particularly the additional SWE and CEUS findings.

Because of the extremely low incidence of breast EMC, the available imaging data on this tumor remain limited. Previous reports have shown that the mammographic findings of EMC are non-specific. It may appear as a newly developed dense mass or nodular opacity with irregular morphology and variable margin characteristics, ranging from relatively circumscribed to indistinct, obscured, or irregular margins; typical malignant calcifications are not commonly observed (10, 11). In the present case, mammography initially classified the lesion as BI-RADS 3 and showed only a suspicious nodular opacity in the upper quadrant of the right breast. Non-contrast chest CT also showed only a breast nodule with peripheral calcifications, without definite malignant features. To date, only one published article has described MRI findings of breast EMC (12). In that report, MRI showed high signal intensity on DWI, heterogeneous enhancement, and malignant enhancement kinetics, and some cases may show internal necrotic areas. In the present case, MRI demonstrated a lobulated mass in the upper inner quadrant of the right breast, with high signal intensity on both T2-weighted and diffusion-weighted images. Dynamic contrast-enhanced MRI showed marked enhancement with a washout-type time–signal intensity curve, which was partially similar to the previously reported malignant enhancement kinetics.

In contrast, ultrasound provided more imaging information suggestive of malignancy. Previous grayscale ultrasound reports have described breast EMC as a solid hypoechoic or mixed-echoic mass, with either irregular or oval morphology and microlobulated margins (13, 14). In the present case, conventional ultrasound showed a mass with heterogeneous internal echogenicity, containing small irregular hypo- to anechoic areas. Multiple punctate hyperechoic foci were observed at the periphery. The lesion had indistinct margins, an overall irregular shape, focal microlobulations, and irregular margins, with slight posterior acoustic enhancement. Color Doppler imaging showed prominent feeding vessels entering the lesion from the periphery. In addition to conventional imaging, SWE was performed in this case and demonstrated heterogeneous stiffness distribution within the lesion, with a higher mean elastic modulus than that of the adjacent normal glandular tissue, suggesting stiffness heterogeneity within the tumor. Similar to previous reports, the sonographic findings in this case suggested several malignant features.

The most noteworthy finding in the present case was the perfusion pattern demonstrated by CEUS. The lesion began to enhance 8 s after contrast injection and reached peak enhancement at 19 s, with a peak intensity of 25.53 dB. Overall, the lesion showed hyperenhancement, followed by washout in the delayed phase, indicating a rapid wash-in and wash-out pattern. The internal enhancement was markedly heterogeneous, with multiple linear hyperenhancing areas in a slightly map-like distribution, interspersed with patchy hypo- to non-enhancing areas. To date, dedicated reports on CEUS features of breast EMC remain lacking. In the present case, the rapid wash-in and wash-out pattern, heterogeneous hyperenhancement, and perfusion defects showed certain similarities to the CEUS features of common malignant breast tumors, such as invasive ductal carcinoma (15, 16), and may provide additional information regarding the perfusion pattern of breast EMC.

Previous CEUS studies of breast cancer have shown that heterogeneous enhancement and perfusion defects are commonly observed in breast cancers with more aggressive pathological features and may be related to uneven tumor vascular distribution, immature neovascularization, local hypovascularity, and relative blood supply insufficiency caused by rapid tumor growth. In the present case, pathological sections showed prominent fibrous stromal components, without definite necrosis. Therefore, the linear hyperenhancing areas on CEUS may be associated with unevenly distributed intratumoral microvascular structures, whereas the hypo- to non-enhancing areas may reflect dense fibrous stroma, locally reduced vascularity, or intratumoral structural heterogeneity. For a rare biphasic tumor such as EMC, the map-like linear enhancement and low-perfusion areas demonstrated by CEUS may provide additional information beyond conventional grayscale ultrasound for visualizing the heterogeneity of internal tissue architecture and microcirculatory perfusion. However, because precise one-to-one imaging–pathology correlation was not performed in this case, the relationship between these CEUS findings and specific pathological components remains speculative and requires further validation in more cases with detailed imaging–pathology correlation. Moreover, these findings are not specific to EMC, and the final diagnosis still depends on histopathological and immunohistochemical confirmation.

## 4 Conclusion

Breast epithelial–myoepithelial carcinoma is a rare biphasic malignant tumor with non-specific imaging findings that may overlap with those of more common breast malignancies. In the present case, multimodal ultrasound, particularly SWE and CEUS, provided additional information regarding lesion stiffness and intratumoral perfusion heterogeneity beyond conventional imaging. These findings may help characterize the biological behavior and malignant potential of the lesion, although they should not be regarded as tumor-specific diagnostic features of EMC. Further accumulation of cases, standardized imaging protocols, and detailed imaging–pathology correlation studies are warranted to better clarify the imaging characteristics of breast EMC and the potential clinical value of SWE and CEUS.

## Data Availability

The original contributions presented in the study are included in the article/supplementary material. Further inquiries can be directed to the corresponding authors.
